# Editorial Comment: Reasons for cessation of clean intermittent catheterization after spinal cord injury: Results from the Neurogenic Bladder Research Group spinal cord injury registry

**DOI:** 10.1590/S1677-5538.IBJU.2021.03.03

**Published:** 2021-03-25

**Authors:** Marcio Augusto Averbeck

**Affiliations:** 1 Hospital Moinhos de Vento Unidade de Videourodinâmica Porto AlegreRS Brasil Chefe de Neuro-Urologia, Unidade de Videourodinâmica, Hospital Moinhos de Vento. Porto Alegre, RS, Brasil.

Darshan P. Patel^1^, Jennifer S. Herrick^2^, John T. Stoffel^3^, Sean P. Elliott^4^, Sara M. Lenherr^1^, Angela P.Presson^2^, et al.

Neurourol Urodyn. 2020 Jan;39(1):211-219.

## COMMENT

The NBRG registry is a prospective observational study examining neurogenic bladder related QoL among individuals after SCI. Participants were recruited throughout the United States and Canada, answering telephonic interviews and electronic questionnaires. Progressive and congenital neurologic disorders were exclusion criteria.

There were 1479 participants who enrolled in this study. Of these participants, 309 (20.8%) identified their primary management as an IDC or a urinary conduit. In these participants, 176 (110 tetraplegic; 66 paraplegic) had used CIC in the past and discontinued this management for SPT (64%), Foley catheter (29.5%), or urinary conduit (6.8%). The most common self′reported reasons for CIC cessation among individuals with SCI were inconvenience, urinary leakage, and too many urinary tract infections.

In the unadjusted comparison, paraplegic participants who had discontinued CIC were older and presented with more comorbidities compared to tetraplegic participants. On the other hand, paraplegic participants had better fine motor scores and performed CIC for a longer duration before cessation, in comparison to tetraplegic participants. Another relevant observation was that a higher proportion of paraplegic participants who discontinued CIC reported chronic pain.

A better understanding of all factors related to poor compliance to intermittent catheterization is certainly needed, as there are inherent implications on the transition to other bladder management modalities. Continuous education by multidisciplinary teams and effective treatment of comorbidities, such as chronic pain, may potentially lead to better outcomes at the long run. Further studies should assess the patients’ view on the most important reasons for discontinuation of intermittent catheterization and the impact of newer catheter technologies on treatment adherence and reduction of complications.

